# Using technology to promote integration of care

**DOI:** 10.1186/1940-0640-10-S1-A20

**Published:** 2015-02-20

**Authors:** David H Gustafson

**Affiliations:** 1 The Center for Health Enhancement Systems Studies, University of Wisconsin-Madison, Madison, WI, 53706, USA

## 

This work focuses on the role that technology can play in integrating addiction, mental health, and medical care services. Behavioral health is being integrated into primary care, spurred by the promise of improved patient outcomes and cost savings. Integration enables providers to care for the whole patient—who often has co-occurring behavioral and physical illnesses. Integration requires change of both organizations and individuals, and technology can play a key role in facilitating both. Technology makes more data available to clinicians and provides tools (such as prediction models, decision-support protocols, and dashboards) to understand clinical data. Among individuals, technology can help patients, family members, and clinicians stay informed and engaged in treatment. Technology can: 1) help identify optimal combinations of interventions for different combinations of conditions; 2) include a greater number of conditions in screening; 3) send reminders and alerts based on data from sensors; and 4) make chronic disease self-management more effective. This study presents a systematic review of the literature on the use of mobile health technology for the treatment of alcoholism; the relationship between utilization of mobile health technology and in-person care; early findings from a project aimed at implementing mobile health technology for addiction; and the role of telemedicine in aiding access to medication-assisted treatment for opioid dependence.

## Trial registration

ClinicalTrials.gov NCT01003119

ClinicalTrials.gov NCT01963234

ClinicalTrials.gov NCT01702142

## Grant support

NIAAA 5R01AA017192-05

NIDA 5R01DA034279-03

NIDA 5R01DA030431-03

NIDA 1R34DA036720-01A1

